# Predicting emergence of crystals from amorphous precursors with deep learning potentials

**DOI:** 10.1038/s43588-024-00752-y

**Published:** 2024-12-18

**Authors:** Muratahan Aykol, Amil Merchant, Simon Batzner, Jennifer N. Wei, Ekin Dogus Cubuk

**Affiliations:** Google DeepMind, Mountain View, CA USA

**Keywords:** Atomistic models, Synthesis and processing

## Abstract

Crystallization of amorphous precursors into metastable crystals plays a fundamental role in the formation of new matter, from geological to biological processes in nature to the synthesis and development of new materials in the laboratory. Reliably predicting the outcome of such a process would enable new research directions in these areas, but has remained beyond the reach of molecular modeling or ab initio methods. Here we show that candidates for the crystallization products of amorphous precursors can be predicted in many inorganic systems by sampling the local structural motifs at the atomistic level using universal deep learning interatomic potentials. We show that this approach identifies, with high accuracy, the most likely crystal structures of the polymorphs that initially nucleate from amorphous precursors, across a diverse set of material systems, including polymorphic oxides, nitrides, carbides, fluorides, chlorides, chalcogenides and metal alloys.

## Abstract (web summary)

This study introduces a^2^c, a computational method that leverages machine learning and atomistic simulations to predict the most likely crystallization products upon annealing of amorphous precursors. The a^2^c tool was demonstrated on a variety of materials, including oxides, nitrides and metallic glasses, and can assist researchers in discovering synthesis pathways for materials design.

## Main

**Fig. 1:**
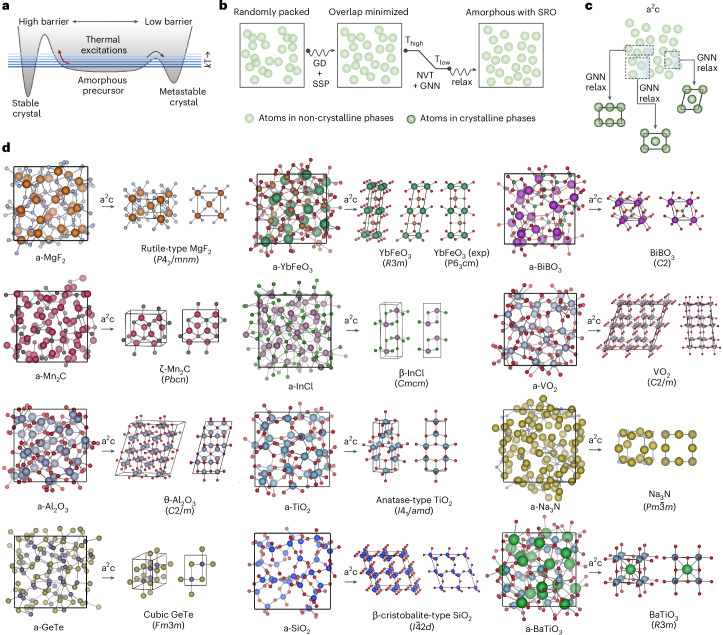
Predicting the crystallization products of amorphous precursors. **a**, Energy landscape showcasing the easier access to an often metastable crystallization product from an amorphous basin compared with a stable crystal due to the lower barrier to nucleation. **b**,**c**, Computational pipeline for generating atomistic models of amorphous atom configurations with properly developed short-range order (SRO) (**b**) and geometric optimization of subcells of various sizes from the amorphous configuration, enforcing periodicity (**c**). **d**, The crystal structures predicted to nucleate from the amorphous materials agree with experiments. Crystals are shown in oblique and side view projections. For YbFeO_3_, the corresponding experimental structure is also shown with the a^2^c prediction due to the stacking sequence variation. NVT, constant volume–constant temperature molecular dynamics; SSP, soft-sphere interatomic potential; GNN, graph neural network-based interatomic potential. GD, gradient descent. Barriers in **a** should be interpreted as generalized barriers encompassing entropic effects^44^. Radial distribution functions corresponding to each of these amorphous precursor and crystal phases are shown in Supplementary Fig. 2. In the respective atomic structures in **d**, the size/color of spheres depicting different types of atom are as follows: Mg, large/brown; F, small/gray; Yb, large/green; Fe, medium/brown; O, small/red; Bi, large/purple; B, small/green; Mn, large/dark magenta; C, small/dark gray; In, medium/purple; Cl, small/green; V, large/gray; Al, large/gray; Ti, large/light blue; Na, large/yellow; N, small/light gray; Ge, medium/dark purple; Te, medium/dark yellow; Si, medium/blue; Ba, large/green. Source data

When amorphous solids crystallize, the first phases to emerge are often not the thermodynamic ground states, but are instead metastable crystals^1–5^. The formation of metastable phases during such crystallization processes is ubiquitous in nature, a well-known example being the crystallization of amorphous calcium carbonate to vaterite, aragonite or calcite, which have roles in biomineralization and the carbon cycle^6,7^. The crystallization sequences of amorphous ice and interstellar molecules have importance in geology, astrophysics and astrobiology^8,9^. In materials science, the amorphous-to-crystalline transformation underpins the development of new technologies, including phase-change memories^10^, nanocrystallized metallic glass soft magnets^11^ and ceramics with finely tuned electronic or optical properties^12^. In synthesis in particular, the crystallization of non-crystalline precursors plays a central role^8,13–15^. The complexity of nucleation has hindered the formulation of polymorph-selective synthesis models^16^, but has encouraged the development of numerous experimental techniques to explore the energy landscape, such as solid-state reactions, chimie douce, deposition, rapid cooling or high-pressure methods^17,18^. Unlike polymorphs, an amorphous precursor can be accessed with many of these techniques and used as a precursor to metastable crystals^13,14,19^. This path to metastable crystals is an instance of the rule of Ostwald, explained by classical nucleation theory, whereby crystals that share local structural motifs with the amorphous precursor will have a lower barrier to nucleation upon annealing (Fig. 1a)^1,20,21^. The structural connection renders the amorphous state a uniquely useful starting point for the predictive synthesis of crystals. To capture the energy landscape itself, state-of-the-art deep learning potentials trained on first-principles calculations are well poised to provide a reliable description at the scale of atoms^22–24^. Guided by the ubiquity of the amorphous precursor and its local structural connection to crystals, here we introduce a computational approach, known as a^2^c, that achieves highly accurate predictions of the (meta)stable crystallization products of amorphous matter with the transformation of local motifs in disordered states into ordered structures, using universal deep learning potentials, at a scale impractical for ab initio methods.

## Results

With a^2^c we start by creating an atomistic model of the amorphous precursor that captures the essential short-range order via melt-and-quench molecular dynamics (MQMD)^25^ (Fig. 1b). We hypothesize that if a large number of subcells of the amorphous atom configuration are relaxed under periodic-boundary conditions following downhill energy gradients using an accurate interatomic potential (Fig. 1c), a fraction of those with the seeding motif should relax into the adjacent basin, which nucleates in practice (as exemplified in Supplementary Fig. 1). The lowest-energy configurations reached with this process should be a close match to the crystal structure of the experimentally observed initial crystallization product of the amorphous material, irrespective of the product being stable or metastable. Initial crystallization products predicted using a^2^c for 12 inorganic systems are presented in Fig. 1d. In each case, a^2^c sifts through tens of thousands of local motifs in the amorphous precursor using deep learning-based interatomic interactions, and identifies the correct crystal structure matching the experimental product (Supplementary Fig. 2 and Supplementary Notes 1 and 2).

**Fig. 2:**
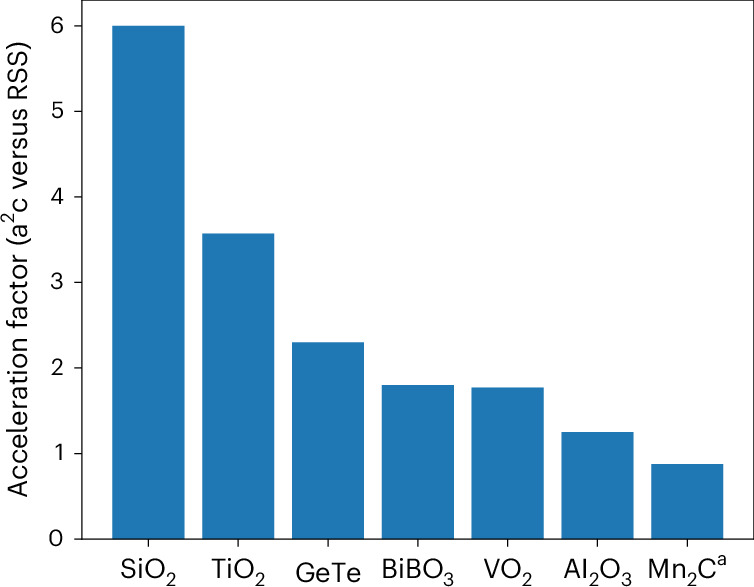
Benchmarking a^2^c in predicting the crystallization products of amorphous precursors. Comparison of a^2^c and RSS in identifying the crystal structure of the crystallization product of amorphous forms of SiO_2_, TiO_2_, GeTe, BiBO_3_, VO_2_, Al_2_O_3_ and Mn_2_C. The acceleration factor is defined as the ratio of the precision obtained with a^2^c to that obtained with RSS. ^a^For Mn_2_C, we could not find the experimental phase with RSS with the present settings. The distribution of crystal structures for each system with each method is shown in Supplementary Fig. 2. See Methods for details. Source data

In terms of utility, a^2^c is complementary to existing crystal structure prediction methods, particularly those that start with randomized initial configurations^26–29^, as it optimizes not for finding a broad range of viable structures, but for selectively finding polymorphs that are realizable in experiments via crystallization of the amorphous precursors. In Fig. 2, this distinction in utility is quantified by computing the ‘acceleration factor’, which is defined as the ratio of the precision obtained by a^2^c to that obtained by a random structure search (RSS)^28^ in finding the actual crystallization products of the amorphous precursors (Supplementary Fig. 3). We find that this factor ranges from ~1.2- to ~6 fold in systems where both methods were able to identify the product. This increase in polymorph selectivity arises from a^2^c confining the search to the part of the energy landscape near the amorphous basin (Fig. 1), whereas RSS, by design, performs a more global structure search.

Encouraged by the examples discussed, we focus next on how a^2^c can complement experimental observations by presenting several case studies.

### Case study 1

The BiBO_3_ system has two metastable polymorphs, (I) and (II)^30^. Polymorph (I) can be accessed by crystallizing the melt, and its structure has been solved using diffraction^31^. Polymorph (II) can only be synthesized by crystallizing the glassy precursor, and its structure remained unsolved until Shinozaki and colleagues^32^ showed that it belongs to space group *C*2. The lowest-energy a^2^c product matches the experimental diffraction pattern, providing a solution for the crystal structure of BiBO_3_ (II) by including atomic positions (Supplementary Fig. 4).

### Case study 2

**Extended Data Fig. 1:**
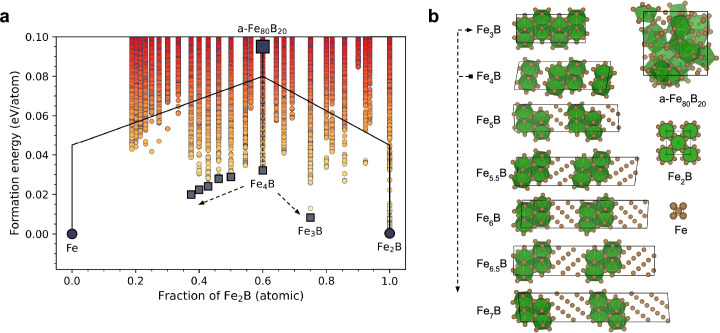
Phase evolution upon crystallization of Fe_80_B_20_ metallic glass as predicted by a_2_c. (**a**) Formation energy of the crystallized phases versus composition, with respect to Fe and Fe_2_B as terminals. No data outside a^2^c outputs are used. (**b**) Atomic structures of the amorphous cell and crystal phases emerged from application of a^2^c (phases highlighted in (**a**) with dark blue markers). Green and brown circles represent B and Fe atoms, respectively. In boron-containing structures, B-centered Fe clusters are shown as green polyhedra. In (**a**), the arrows highlight the decomposition pathway of Fe_4_B towards phases highlighted in dark blue markers, where compositions farther away from the overall 4:1 ratio appear later in annealing during experiment. Coloring is a guide for the eye that scales with formation energy. Amorphous Fe_80_B_20_ energy falls outside the shown range and is repositioned for visual purposes. Source data

The initially crystallizing metastable phases often have compositions close to those of their amorphous precursors, because decomposition into multiple phases with diverging compositions would be penalized by solid-state transport. Under such conditions, we anticipate decomposition of the well-known metallic glass a-Fe_80_B_20_ to fan out gradually to nearby metastable crystals^5^, which is found to be consistent with the phase landscape obtained using a^2^c (Extended Data Fig. 1). Our results show that crystallization of a-Fe_80_B_20_ involves topotactic decomposition to a series of related phases towards Fe and Fe_3_B, in accord with the spinodal-like phase separation suggested for such glasses^5,33,34^. The a^2^c predictions reconcile the available experimental knowledge into a phase decomposition pathway for Fe_80_B_20_ (Supplementary Note 3).

### Case study 3

**Extended Data Fig. 2:**
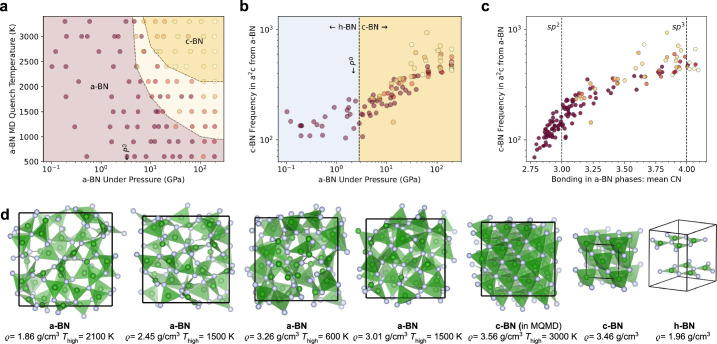
Polymorph selection through amorphous precursor morphology in boron nitride. In panels (**a**)–(**d**), a-BN, c-BN and h-BN denote amorphous, cubic and hexagonal phases of BN, respectively. (**a**) Morphology map of structures generated using MQDM as a function of quench temperature and pressure, where circles are colored on a scale ranging from red to yellow corresponding to MQMD runs where the structures in repeating simulations at each parameter combination were 100% amorphous or 100% c-BN, respectively (further details in Supplementary Methods). The shaded regions outline amorphous, and partially and mostly crystalline regions. (**b**) Frequency of the appearance of subcells that converted into c-BN in a^2^c for each parent phase shown in (**a**) as a function of pressure on a-BN or partial or c-BN phases [color coding identical with (**a**)]. P^0^ denotes the computationally estimated equilibrium pressure for h ↔ c transition at zero temperature. (**c**) The same frequency in (**b**), plotted against the mean B-N coordination number (CN) in the parent MQMD phases. (**d**) Sample atomic structures of a-BN and c-BN phases that emerged from MQMD, along with the ideal c-BN and h-BN phases. B and N atoms are shown as green and gray spheres, respectively. B-N coordination environments are highlighted with green polyhedra. Source data

Polymorph selection by changing the morphology of an amorphous precursor is a promising but challenging research direction. Synthesis of *sp*^3^-hybridized cubic boron nitride (c-BN) from the *sp*^2^-hybridized hexagonal polymorph (h-BN) requires excessive pressures and temperatures, whereas starting with amorphous BN (a-BN) requires less extreme conditions^35–37^. We find that the fraction of nominally a-BN subcells that crystallize into c-BN from a given a-BN cell increases with pressure, which is explainable by the continuous shift from *sp*^2^ to *sp*^3^ in B–N bonding in a-BN with pressure (Extended Data Fig. 2 and Supplementary Fig. 5).

Anatase and rutile are the two polymorphs a^2^c predicted to selectively form from a-TiO_2_ (Supplementary Fig. 3). With further analysis, we find that the frequency of subcells that crystallize into anatase versus rutile in a^2^c is influenced by the local ordering of TiO_6_ octahedra in amorphous TiO_2_ (Supplementary Figs. 6–8 and Supplementary Note 4). These findings agree with the sensitivity to the preparation conditions of the crystallization products of amorphous oxide films of multivalent cations such as Ti^38–40^. We further confirm that the findings are consistent with all-atom simulations sampling amorphous–crystalline TiO_2_ interfaces (Supplementary Figs. 9 and 10 and Supplementary Note 5), where anatase has the lowest interfacial energy with a-TiO_2_, and rutile remains competitive. Unlike a^2^c, however, such interface simulations would quickly become impractical to run at scale for unexplored systems (Supplementary Methods). Nevertheless, a^2^c is not a replacement for all such simulations. For example, as a limitation, sensitivity of the product to oxygen substoichiometry or thickness in thin a-TiO_2_ films, and subsequent appearance of brookite^38,40^, could not be captured by a^2^c and requires simulations of the deposition and nucleation to understand (Supplementary Note 6). In other words, although the melt–quench used here yields reasonable amorphous configurations to apply to a^2^c and it is possible to vary the morphology to some degree with quench rate or density, the actual experimental structure of the amorphous precursor can vary based on the synthesis process, and an explicit modeling of that process itself may be needed in certain cases^41^. Similarly, capturing transitory phases on the path to the product, as in a-MgF_2_ (refs. ^42,43^), or understanding the real-time crystallization pathway of a-GeTe^44^ requires all-atom simulations of the transformation.

## Discussion

On the atomic level, the size of the atom displacements involved in the transformations observed with the a^2^c approach, from the amorphous precursor to atom arrangements containing ordered structures, is reminiscent of the atomic shifts occurring in displacive and dilatational polymorphic phase transitions in Buerger’s classification^18,19^, which take place between polymorphs with structural relationships under limited mobility (for example, crystallization at lower temperatures). Unlike the reconstructive cases, these transitions require minimal bond breaking or rearrangement beyond distortions or rotation, and they occur via a low-energy interface due to the structural similarity of the involved polymorphs. Accordingly, these transitions proceed rapidly^18^, and any nucleus can grow easily into its neighborhood in the amorphous matrix. This mechanistic nature has parallels with what is modeled atomistically with a^2^c, hence explaining why its predictions are consistent with experimental observations in many different chemical spaces.

Our work has demonstrated that prediction of the products of amorphous-to-crystalline transformations with atomic-level explainability is possible by combining a local motif search with the general-purpose deep learning interatomic potentials in many inorganic systems. Predictive agreement with experimental polymorphs across a wide range of chemical spaces validated the computational framework. With this validation, the presented approach can be employed in research tasks such as resolving the outcome of such transitions in complex material systems, or in the in silico discovery of material polymorphs or glass-crystal composite systems that inherently have synthetic pathways starting from the amorphous state.

## Methods

### Generation of amorphous structures

The MQMD process in a^2^c, shown in Fig. 1b, aims to efficiently obtain reasonably representative amorphous configurations in a three-stage procedure. In the first stage, the *N* atoms comprising the system are distributed randomly in a cubic box. Except when explicitly modeling variations in density, the density of this box is determined heuristically with a rule of thumb of 15% (adopted from the survey by Cui and colleagues^45^) volumetric expansion of the volume per atom of the known stable phase or averaging the same in the case of the presence of a phase mixture of that composition on the convex hull in our standard workflow. This rule of thumb is similar to those previously reported in the literature^25^ and is reminiscent of the Lindemann criterion^46^, and it yields consistent amorphous configurations with no cavities. To maintain throughput and efficiency, *N* is restricted to the exact integral representation of the composition closest to 100, which has been shown to yield sufficiently representative structures in inorganic systems^25^. The random atomic positions are then briefly relaxed with a soft-sphere interaction potential to minimize the atomic overlaps. We used the soft-sphere interaction in JAX-MD^47^, which includes an empirical quadratic repulsion at distances shorter than a predefined distance, and is zero elsewhere. This preparation stage ensures that the subsequent amorphous structures do not inherit fingerprints from melting of any particular crystal; instead they rapidly develop the proper short-range structural elements inherent to liquid and amorphous configurations in the equilibration and quench stages^25^. The number of steps in overlap minimization is limited to 30, or stopped early when the shortest interatomic distance reaches above the 90% of summed ionic radii of the atoms of each pair. Limiting the number of steps in this process ensures that we do not inject any arbitrary local arrangement using the soft-sphere potential. The next stage in the workflow is a three-step MD protocol in which a graph neural network (GNN) force field is used throughout: equilibrate a melt at *T*_high_, cool/quench this phase to *T*_low_, and equilibrate at this temperature. *T*_high_ and *T*_low_ were manually selected for each system (Extended Data Table 1), and we ran each of the three stages of the protocol for 1,500, 2,500 and 1,500 steps, respectively. The term ‘equilibrate’ refers to constant-temperature MD runs and does not imply that an equilibrium state is reached (which would be particularly impractical and irrelevant for simulations mimicking low-temperature deposition). In MD, we use the *NVT* ensemble with a Nose–Hoover thermostat and a timestep of 2 fs. The atomic positions can be relaxed with a short gradient descent (GD) run in the last stage; this was kept optional as we did not find any impact on crystallization. The MQMD workflow is implemented in JAX-MD^47^ and can be run in high throughput^48^. The representativeness of the amorphous structures generated by our MQMD workflow was validated by comparing with experimental data in several systems (Supplementary Fig. 11 and Supplementary Note 7).

### Crystallization workflow

The crystallization phase in a^2^c (Fig. 1c) accepts the amorphous structure model generated in the MQMD as input, and exhaustively creates all orthorhombic parallelepipeds (subcells) that can be generated from this structure, keeping their edges parallel to the unit vectors of the parent cubic box (an early example of a subcell-finding approach is provided in ref. ^26^). These subcells are allowed to be of any length and at any position on the *n*_grid_ × *n*_grid_ × *n*_grid_ point net, where the first point is located at position (0, 0, 0), encompassing the parent structure. Our default for *n*_grid_ is 10, except for Fe_80_B_20_, as noted in Extended Data Table 1. The full grid has ~1.7 × 10^5^ subcells for *n*_grid_ = 10 and ~1.0 × 10^7^ subcells for *n*_grid_ = 20. Because all the degrees of freedom of each subcell are allowed to relax (as described later), we found that the present settings were able to yield a comprehensive enough set of subcells to search for crystal seeds in the systems studied here, even for finding non-orthorhombic products. However, in any system, this algorithmic step can be enhanced by adding additional degrees of freedom to the subcell creation, for instance by allowing triclinic cells or shifts.

These subcells can be filtered further using maximum atom counts per cell or stoichiometry constraints in cases where compositions distant from the parent amorphous starting material are not of interest or not plausible. For example, in Fe_80_B_20_, all subcells are kept up to an atom count of 16, whereas for MgF_2_, subcells having this target stoichiometry and up to 21 atoms are kept. With the stoichiometry and atom count constraints, the subcell set size per system often falls in the range of 10^3^–10^4^ for most systems with *n*_grid_ = 10, and is one to two orders of magnitude larger for *n*_grid_ = 20. Details on each system studied are provided in Extended Data Table 1. Next, all geometric degrees of freedom of these subcells, including atomic positions and the cell size and shape, are optimized using an NequIP-based equivariant GNN potential (see ‘Deep learning methods’) to describe the interatomic interactions, as well as the fire algorithm^49^ for at least 150 steps. The lowest-energy structures found by this procedure at each composition are the selectively predicted stable or metastable crystallization products, which should match the experiments. Matching is confirmed in each case, first by a combination of visual inspection and pymatgen’s structure matching and/or space group analyzer methods^50^, at various tolerance settings. Our lowest-energy bound is strict; that is, the experimental crystallization products were either the absolute lowest-energy a^2^c predictions or practically degenerate within a few milli-electronvolts per atom, which is on par or lower than the variability in density functional theory (DFT) convergence and/or structure optimization. For the studied systems, we provided the generated amorphous structure, the subcells extracted using a^2^c, the index of a particular initial subcell from the amorphous configuration that subsequently relaxed into the target phase, the final force-field structure, and, for further validation, a DFT-obtained final structure of the exact relaxation path starting from the given initial subcell. DFT relaxations were performed with MP2020 settings^51^ (see ‘Data availability’).

### Deep learning methods

General-purpose machine learning interatomic potentials for various deep learning architectures are emerging to model condensed phases^22–24,52–54^, with certain architectures being chemically universal (that is, a single potential is applicable to the entire periodic table and under a variety of modeling conditions). The particular instance of the general-purpose (commonly referred to as ‘universal’) deep learning potential used in this work was developed as part of the GNoME project^24^. The potential adopts the equivariant GNN architecture of NequIP^55^, and uses three layers of message passing, even irreps up to *l*_max_ of 2 with multiplicities of 64, 32 and 16 for *l* = 0, 1 and 2, and a two-hidden-layer radial multilayer perceptron with 64 neurons acting on a radial basis of eight Bessel functions. We used a local interatomic cutoff radius of 5 Å in graph generation. This particular model has ~2.4 million parameters trained on energies and forces, with a loss function composed of equally weighted Huber losses where the energy term is normalized per atom and the force term is normalized by the total number of force components, 3*N*, each with a Huber-loss delta of 0.01. The potential was trained using the Adam optimizer with a batch size of 64 structures and a learning rate of 5 × 10^−3^ using a linear learning rate decay. We trained our potential on a dataset of ~7.4 million DFT calculations. The dataset is made up of structures across a wide array of chemical spaces, sampled from ab initio random-search-generated structures^28^ and substitutionally generated structures as described in the paper outlining the GNoME project^24^. From the substitution data, the first, second and twentieth steps of each relaxation trajectory are sampled. The particular model reaches a test error of 0.04 eV per atom on the MatBench Discovery benchmark^56^. We further showed that this potential is robust in modeling amorphous systems, achieving a testing error of 38 meV per atom with respect to DFT for thousands of amorphous structures encompassing nearly all binary chemical systems^48^. We found this potential to be accurate in predicting the relative stabilities of polymorphs in the chemical systems relevant to this work, with an estimated error of 9 meV per atom and a stability ranking correlation coefficient of 0.8 or above (Supplementary Fig. 12 and Supplementary Note 8). This same potential was used in all the results reported in this work, including both MQMD and crystallization workflows. All MQMD and crystallization simulations were run on a single P100 or A100 graphics processing unit.

### Crystal structure prediction with random structure search

Global exploration methods that utilize random configurations as the starting points of their complex search procedures have a long history in crystal structure prediction^26,28,29,57,58^. Here we used a recent and widely adopted version—random structure search (RSS)—and the respective AIRSS package developed by Pickard and colleagues^28^ to generate around 10,000 structures with bond-distance constraints at target compositions, and relaxed them with our force field. We applied a two-step process to categorize the resulting structures. First we checked if they could be matched to one of the known polymorphs that may nucleate from the amorphous atom configurations using pymatgen’s StructureMatcher^50^, and, if not, we categorized them with respect to their space group. Precision was calculated as the true positive count (in this case, this value is 1 if the known crystallization product is found by the method, or 0 otherwise) divided by the number of all unique polymorphs found within the energy threshold. We used an energy threshold of up to 0.05 eV per atom of the lowest-energy crystal in each system when counting polymorphs. For TiO_2_, the number of unique polymorphs found with RSS is roughly similar to the earlier machine learning-based RSS work by Reinhardt and colleagues^59^.

### Structural analysis and post-processing

Pymatgen was used extensively to process and analyze amorphous and crystal structures^50^. VESTA was used to generate the structure visuals^60^. For smoothness, radial distribution functions (RDFs) and the bond angle distributions for the amorphous structures were averaged over the last 200 steps of the low-temperature equilibration run, in ten-step increments.

## Supplementary information

Supplementary Information Supplementary Figs. 1–12, notes and methods.

## Source data

Source Data Fig. 1 Source data for Fig. 1.

Source Data Fig. 2 Source data for Fig. 2.

Source Data Extended Data Fig. 1 Source data for Extended Data Fig. 1.

Source Data Extended Data Fig. 2 Source data for Extended Data Fig. 2.

## Data Availability

Amorphous structures, the a^2^c subcells extracted from these structures, the indices of one or more initial subcells from the amorphous phase that relaxed into the target phase(s), the final force-field-relaxed structure of the target and the DFT-relaxed structure of the same a^2^c subcell are available at https://github.com/google-deepmind/materials_discovery ref. ^61^. Source data are provided with this paper.

## Extended data

**Extended Data Table 1:** Settings and statistics for the studied systems

System	*T*_high_ (K)	*T*_low_ (K)	Volume (Å^3^/atom)	*n*_max_	Stoichiometry constraint	Subcells relaxed with GNN
MgF_2_	900	150	12.50	21	Yes	1.5 × 10^4^
YbFeO_3_	2500	300	12.39	24	No	3.5 × 10^3^ / 7.1 × 10^4^
BiBO_3_	3000	300	15.58	24	No	5.1 × 10^3^ / 7.3 × 10^4^
Mn_2_C	2500	400	11.37	21	No	1.6 × 10^4^ / 5.5 × 10^4^
InCl	500	75	33.98	20	Yes	1.8 × 10^4^
VO_2_	1500	400	11.86	16	No	1.8 × 10^4^ / 8.8 × 10^4^
Al_2_O_3_	1500	400	10.05	16	No	7.3 × 10^3^ / 9.1 × 10^4^
TiO_2_	2500	400	12.16	16	No	2.4 × 10^3^ / 6.4 × 10^4^
Na_3_N	75	75	29.74	16	Yes	9.6 × 10^3^
GeTe	1150	400	32.35	16	No	9.6 × 10^3^ / 3.3 × 10^4^
SiO_2_	3000	300	18.15	24	Yes	1.1 × 10^4^
BaTiO_3_	3000	300	15.59	30	Yes	4.8 × 10^3^
Fe_80_B_20_	900	400	10.21	16	No	- / 3.9 × 10^6^ (*n*_grid_ = 20)
BN	Range	350	Range	8	Yes	3.1 × 10^6^

If a stoichiometry constraint is applied in subcell selection, only the count of such subcells are shown in the last column. If no stoichiometry constraint is applied, the counts for both the relaxed subcells with target composition (that is the same composition as the amorphous precursor phase) and all relaxed subcells (that is with any stoichiometry) are shown in the last column.

## Code availability

The code for the a^2^c tools and an end-to-end python workflow for running a^2^c are available at https://github.com/jax-md/jax-md ref. ^62^.
