# Supplementary material for: Predicting emergence of crystals from amorphous precursors with deep learning potentials
Source: Nat Comput Sci. 2024 Dec 18;5(2):105–11. doi: 10.1038/s43588-024-00752-y (PMC11860218; doi:10.1038/s43588-024-00752-y)
Supplement: Supplementary file 1 — Supplementary Figs. 1–12, notes and methods. [file 43588_2024_752_MOESM1_ESM.pdf]

nature computational science

Supplementary information

https://doi.org/10.1038/s43588-024-00752-y

Predicting emergence of crystals from amorphous precursors with deep learning potentials

In the format provided by the authors and unedited

Table of contents

Supplementary Methods	2
Supplementary Figures	4
Supplementary Notes	14
Supplementary References	20

Supplementary Methods

Boron nitride search

To identify the effect of quench temperature (T_{high}) and applied pressure (P) on the morphology of the *targeted* amorphous BN precursor generation, and the resulting crystallization process, we ran MQMD workflow over a grid of 15 densities (ϱ) between 1.70 and 4.35 g/cm^3 and 10 T_{high} values between 600 and 3300 K, with up to 5 repeats with different seeds for each each (P,T_{high}) combination. In total, ~715 MQMD simulations were run for BN. Resulting structures were inspected manually to check if structure is amorphized, or if there are occurrences of partial or full crystallization. Pressure for each condition is calculated by averaging that of its repeats. Under extreme pressure and temperature conditions, c-BN appeared even in the MQMD stage (Extended Figs 2a and 2d). Coordination numbers were determined using Brunner's reciprocal gap method.[1,2] For each (P,T_{high}) combination, one of the resulting structures was passed on to the crystallization workflow (Fig. 1). Each of these 150 crystallization runs processed around 20,500 subcells embedded in their parent structure, hence the total number for BN reached ~3.1 million structure optimizations using the GNN potential. For the reference 0 K equilibrium pressure for *h* ↔ *c* transition, which is still under debate,[3] we used the energy difference between *h* and *c* phases as calculated with density functional theory[4] and a simple linear approximation to the PV term in enthalpy,[5] which agrees reasonably with the latest diffusion Monte Carlo calculations[3] and is shown as a guide for the eye. We refer to all disordered BN structures as a-BN, including those that display a turbostratic sp^2 nature (e.g. ϱ = 1.86 g/cm^3 and T_{high} = 2100 K in Extended Fig 2d).

Generation of realistic atomistic models of amorphous-crystalline interfaces

For each polymorph, the workflow for simulating the amorphous-crystalline interfaces starts by generation of stoichiometric slabs of the polymorph with various terminations including all symmetrically distinct planes up to a Miller index of 2, including Tasker II modifications, and searching cells as orthogonal as possible to the **a** x **b** plane considering up to 3 linear combinations of lattice vectors, ensuring slabs to be exclusively thicker than the set minimum of 7 Å using pymatgen.[6–8] Each slab is then enlarged by replication in **a** and **b** (in plane) directions until |**a**| and |**b**| are at least 8 Å. Next, for each slab, amorphous precursor models that are commensurate with **a** and **b** vectors in the base plane of the interface, and are at least 9 Å thick in **c** direction are prepared separately using the MQMD workflow. Since the amorphous material has a statistical nature to it, we generate 10 unique such amorphous structures per termination by initializing the MQMD workflow with different seeds. The crystal slabs are then combined with

their corresponding amorphous precursors in the same simulation cell, where we further sample several random terminations from each of the 10 amorphous precursors prepared for a termination. The sampling addresses the inherent stochasticity in the amorphous configuration and also serves as a mechanism for searching for low energy interfacial conformations. Simulation cells that are larger than approximately 225 atoms are discarded for computational efficiency. Effectively, we combinatorially generate and simulate 2100, 2500 and 1135 amorphous-crystal interface supercells for anatase, rutile and brookite, respectively. To help reconstruct interfacial conformations without disrupting the bulk phases, we run a short NVT-MD simulation at 500 K for 500 steps. For each unique crystal plane, the final interface energies are estimated as the minimum energy found among the different samples of the given *hkl* plane supercells:

$$\gamma_{hkl}^{a||p} \approx min\left\{\gamma_{1,\,hkl}^{a||p},\ \gamma_{2,\,hkl}^{a||p},\ \dots\ ,\gamma_{n,\,hkl}^{a||p}\right\}$$

where for each sample cell *i* we have:

$$\gamma_{i,\,hkl}^{a||p} = (E_{i,hkl}^{a||p} - E_{i,hkl}^{a} - E_{i,hkl}^{p})/2A_{i,hkl}^{a||p}.$$

Here *a* and *p* denote amorphous and polymorph phases, || denotes the interface-containing supercell, *A* is the interfacial area, and *E* are the total energies of respective cells containing the phases in the superscripts.

Titanium dioxide search

To investigate the relationship between atomic structure of the amorphous TiO_2 precursors and their crystallization products, we ran a^2c campaigns where the initial amorphous starting materials were generated with different melt quench times (2, 20, 200 and 2000 ps) and melt-equilibration temperatures (1500, 3000 and 5000 K) in MQMD. For each parameter setting, we performed 10 independent MQMD simulations with different seeds to generate unique amorphous samples. For the quench time scans, we set T_{high} as 1500 K and in temperature scans, we set the quench time as 2 ps, and set T_{low} = 300 K for both scans. Rest of the MQMD and crystallization settings are identical to those in Extended Data Table 1 for TiO_2. For each of the 10 independent amorphous samples per condition, we performed crystallization workflows in a^2c. A Savitzky-Golay filter of window length four and order one were applied to smoothen the RDFs.

Supplementary Figures

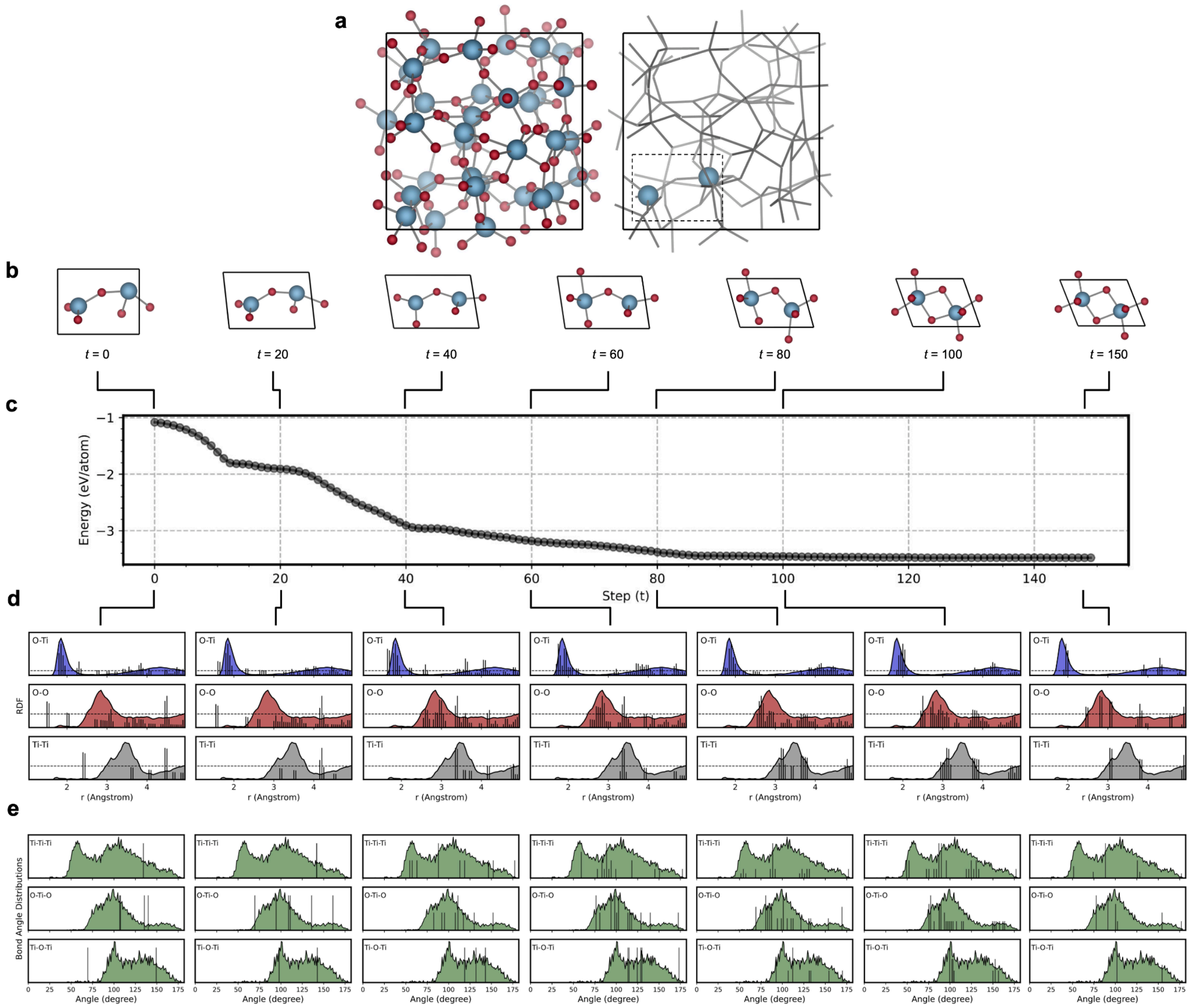

Supplementary Figure 1. Emergence of anatase from a local motif in amorphous TiO_2. (a) The amorphous structure for TiO_2 obtained via MQMD, the first panel showing the entire atomic structure and the second one highlighting the subcell (and its two Ti atoms) being tracked. Evolution of the geometry (b) and energy (c) of the subcell in (a) upon optimization using the GNN-potential under the periodic-boundary constraint leading to the primitive anatase cell. The radial and bond angle distribution functions of the subcells in (b) are shown in (d) and (e) as vertical solid lines, respectively, both overlaying the same functions of the parent amorphous precursor. Intensities are scaled to the same ranges for amorphous and crystal functions for visualization. For consistency across amorphous and crystalline systems, Ti-O, O-O and Ti-Ti maximum bond lengths were approximated as the first minima of the RDF as 3.5, 2.5 and 4.0 Å, respectively. See Supplementary Note 2 for further discussion.

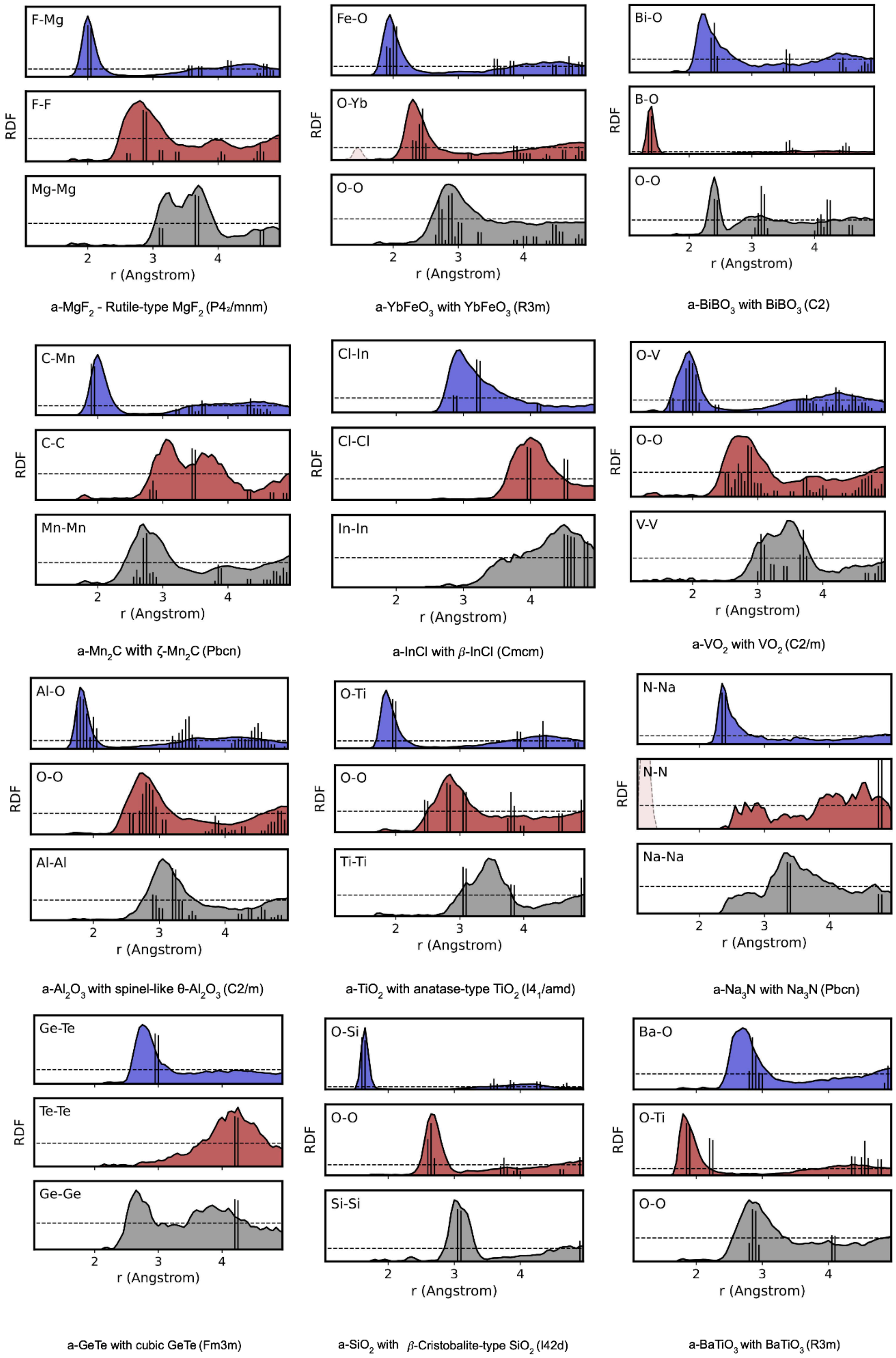

Supplementary Figure 2. Local order in amorphous precursor and crystalline phases. Radial distribution functions (RDFs) are plotted for amorphous precursors shown for the systems in Fig. 1 along with the same function of their initial crystallization products.

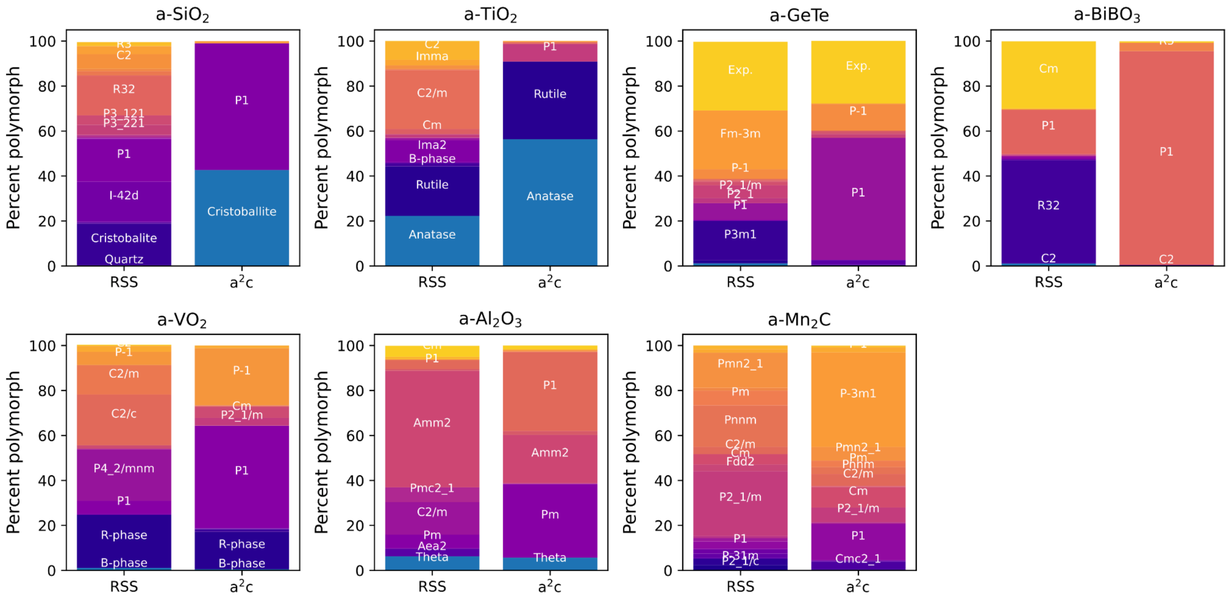

Supplementary Figure 3. Distributions of crystal structures obtained with a^2c and RSS. Structures with more than 2% occurrence or and those with a conventional name are labeled. As expected from a global exploration method, RSS successfully finds many unique and thermodynamically nearly-stable phases, whereas a^2c more selectively finds phases known to crystallize from these amorphous precursors under experimental conditions discussed in the main text. See Methods for details.

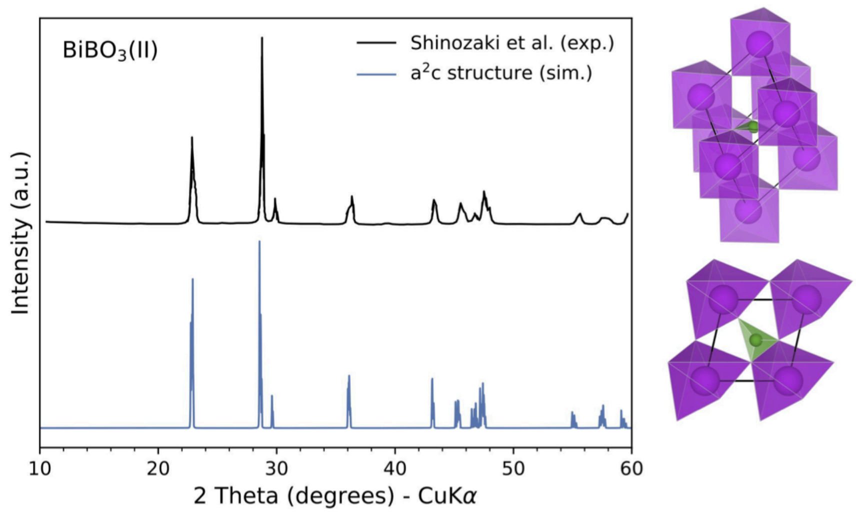

Supplementary Figure 4. XRD pattern of a²c-predicted *C2* $BiBO_3$ structure compared to experiment. The experimental XRD pattern was extracted from Shinozaki et al.[9] manually by image processing. Polyhedral views of the crystal structure are also shown as inset, where purple polyhedra are [BiO_6] clusters, green triangle is the [BO_3] units and O-atoms are not displayed for clarity.

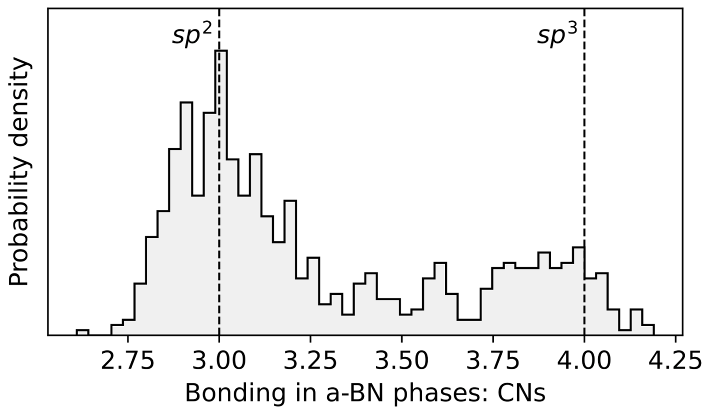

Supplementary Figure 5. Coordination numbers in a-BN. Distribution is calculated from the amorphous BN configurations obtained with the MQMD process as described in Methods.

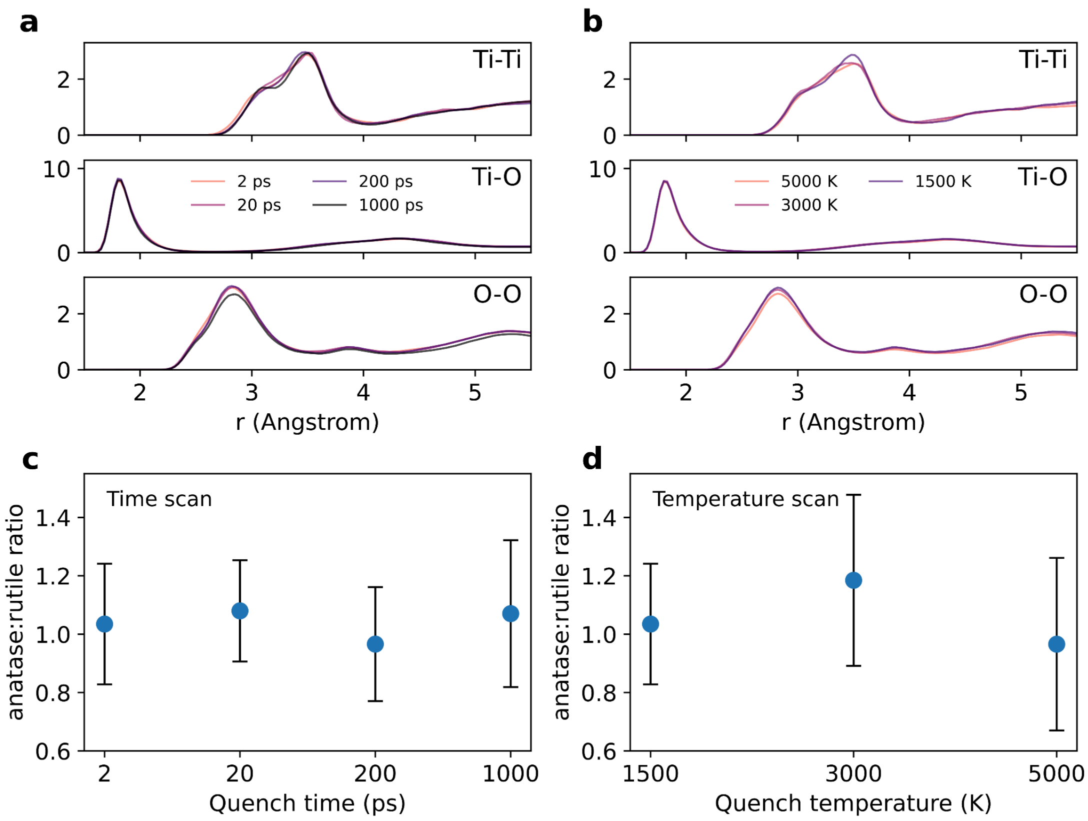

Supplementary Figure 6. Partial radial distribution functions (RDFs) of amorphous TiO_2 precursors prepared under varying conditions in MQMD (a)-(b) and anatase to rutile ratios among crystallization products (c)-(d). Both RDFs and product ratios show the mean values obtained across 10 unique samples (both MQMD and subsequent crystallization) for each respective condition. In (c) and (d), time scans are shown for quench temperature (T_{high}) of 1500 K and temperature scans are shown for 2 ps quench times, respectively. Error bars show one standard deviation. See Supplementary Methods for details.

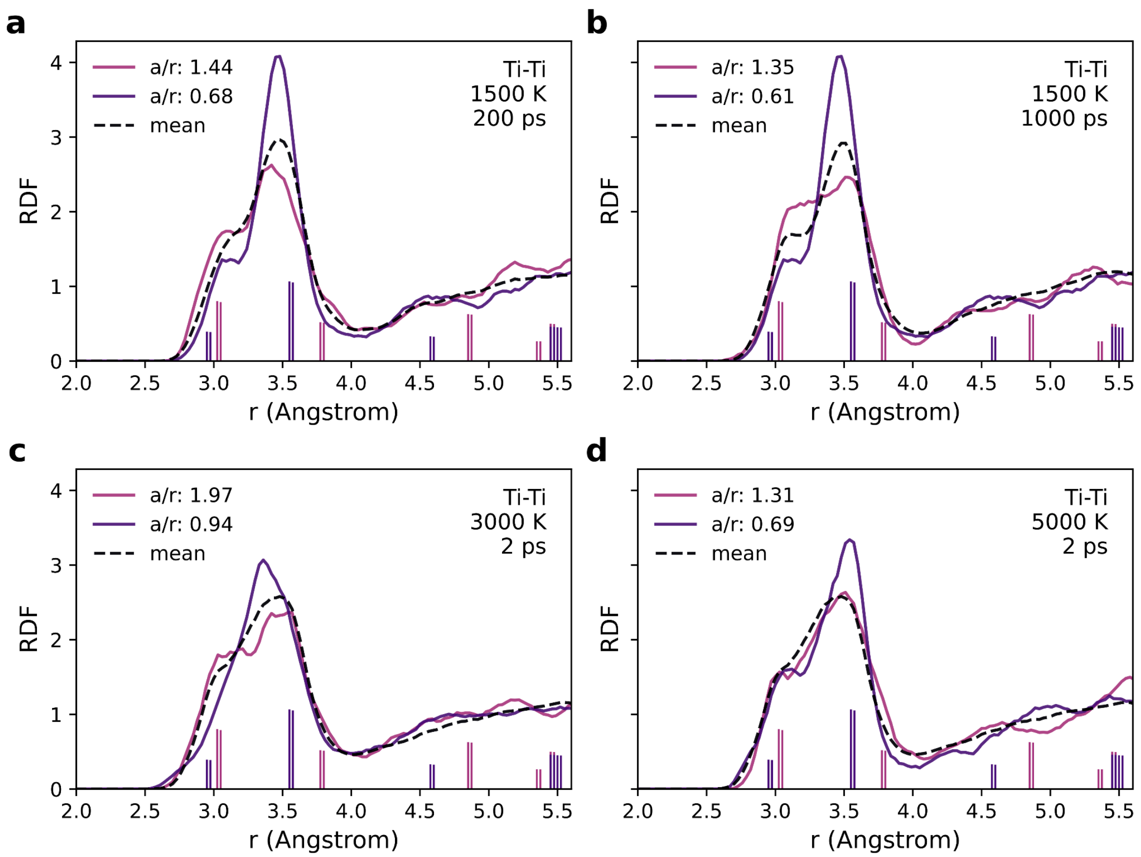

Supplementary Figure 7. Partial Ti-Ti RDFs for individual amorphous samples that led to relatively higher and lower anatase:rutile ratios (“a/r”), at the distinct amorphous sample preparation conditions shown as insets. The mean RDF across all amorphous samples of each condition are also shown. Pink and purple vertical lines represent anatase and rutile, respectively (scaled to fit the plots). See Supplementary Methods for details.

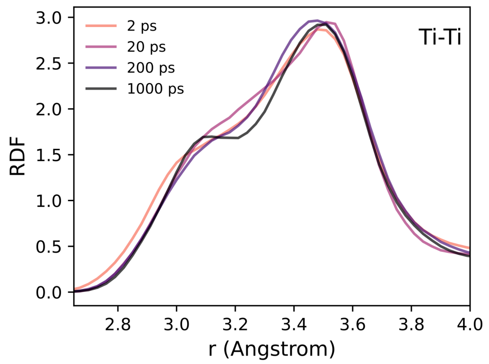

Supplementary Figure 8. A closer view of the mean Ti-Ti RDFs shown in Supplementary Figure 5a.

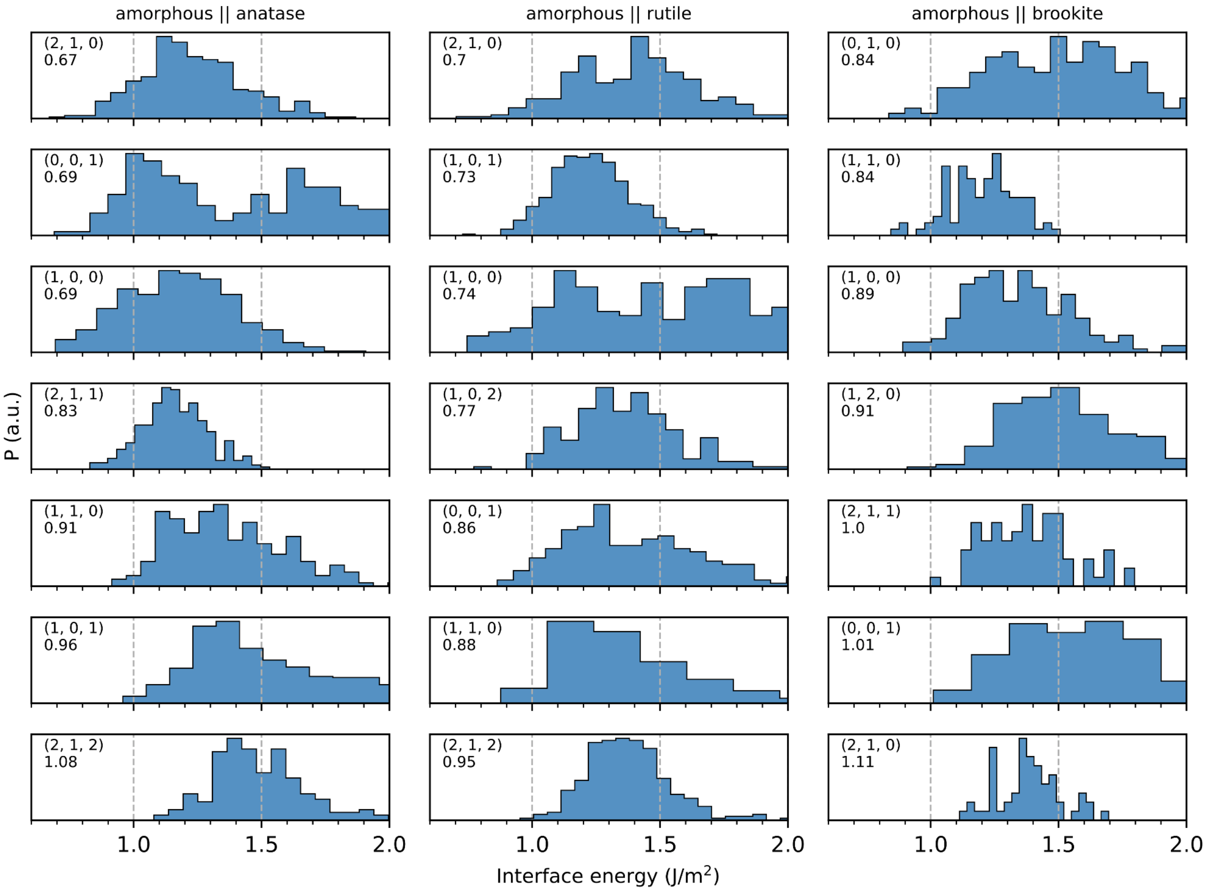

Supplementary Figure 9. Distributions of interface energies of various terminations in amorphous || polymorph interface supercells for TiO_2. Miller index and lowest interface energy (in J/m^2) of the particular plane termination are shown in the top left corners of each panel. For rutile (1,1,2) and (1,1,1) planes, both of which were found to have 0.96 J/m^2 energy minima are not shown for visual purposes.

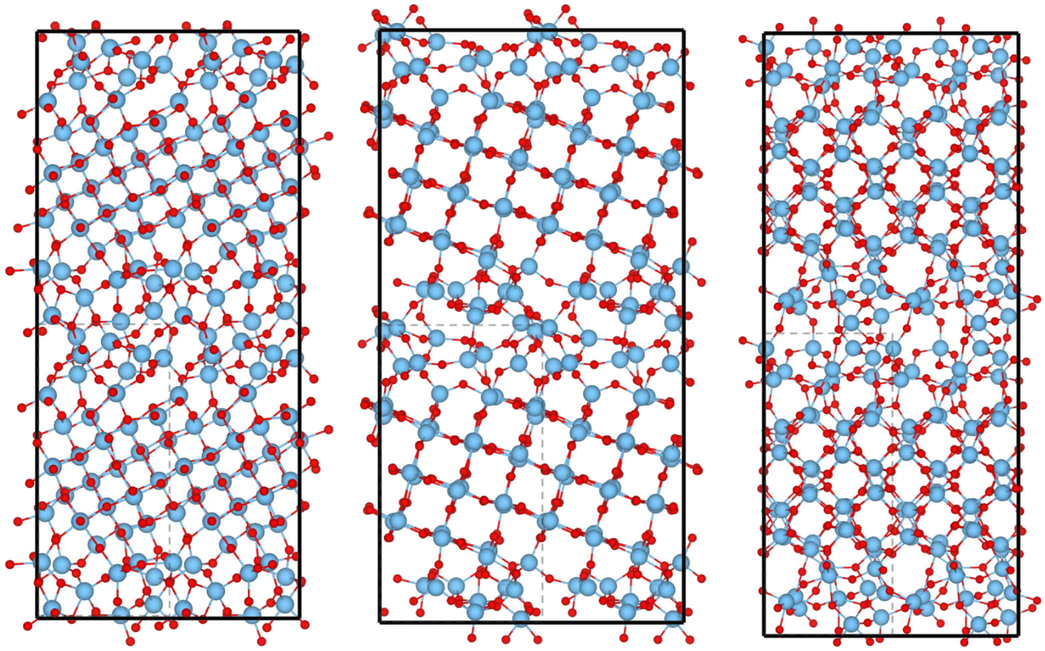

Supplementary Figure 10. Low energy amorphous film || crystalline polymorph interface supercells for (2,1,0)-anatase, (2,1,0)-rutile and (0,1,0)-brookite TiO_2, from left to right. For uninterrupted visualization of each system across interfaces, 2x2x2 replications of the original supercells (dashed lines) are shown.

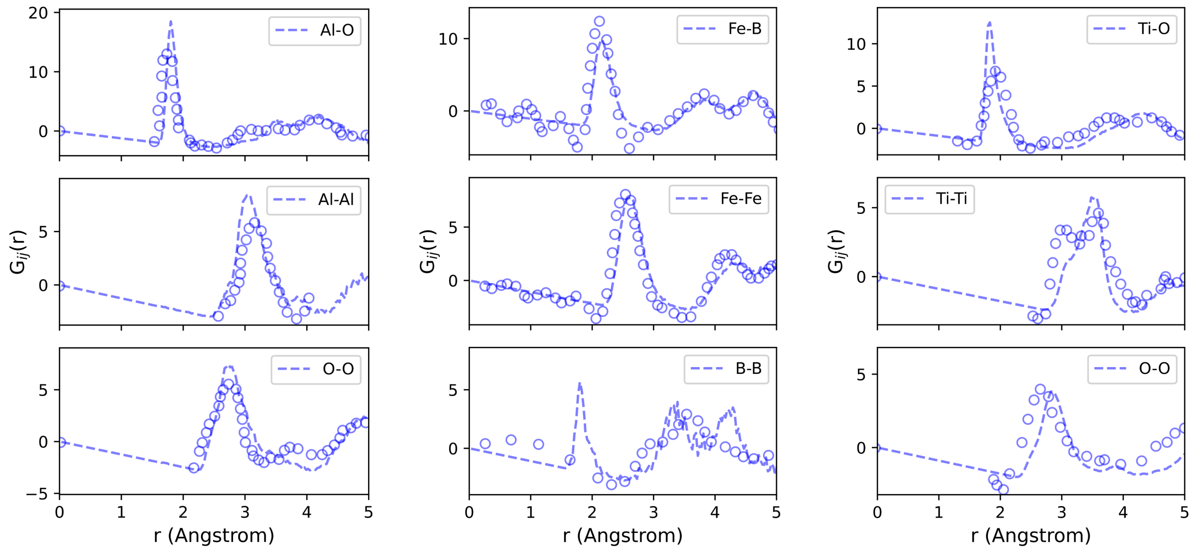

Supplementary Figure 11. Reduced partial radial distribution functions calculated from the amorphous structures in this work (dashed lines) compared to those derived from diffraction measurements reported in the literature (circles) for amorphous Al_2O_3, $Fe_{80}B_{20}$ and TiO_2.[10–12] The experiment-derived RDFs are extracted manually from the figures in respective publications. See Supplementary Note 7 for a discussion of the agreement between current and experiment-derived RDFs.

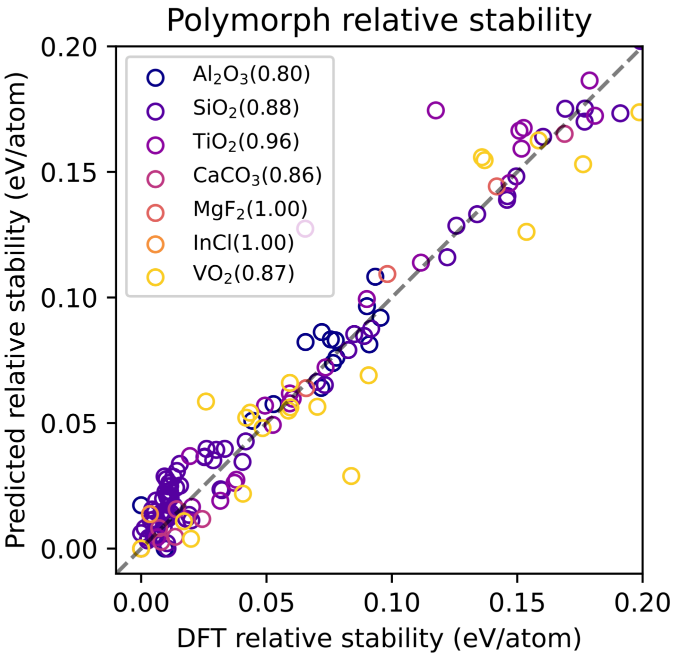

Supplementary Figure 12. Relative stabilities of polymorphs predicted by the GNN potential used in this work, compared to the DFT values for seven polymorphic systems. Spearman's rank correlation coefficient for each system is shown next to the chemical formulas in the legend. Further details are explained in Supplementary Note 8.

Supplementary Notes

Supplementary Note 1

Metal oxides, given their abundance in nature and as subjects of broad technological interest, offer well-studied polymorphic systems with known crystallization sequences. In the case of amorphous (labeled as “a”) TiO_2, the first crystallization product is often anatase.[13,14] Before reaching quartz, a-SiO_2 visits various polymorphs, the first being cristobalite.[15] On crystallization of a-VO_2, the less common B-phase appears initially, prior to the ground-state R-phase.[16] Ebralidze et al.[17] found that when the anomalous expansion of a-$BaTiO_3$ films[18] is suppressed, they crystallize into cubic $BaTiO_3$. In all these cases, a^2c correctly finds the initial crystallization product of the amorphous starting materials.

Metal halides, carbides, nitrides and chalcogenides possess different levels of ionic and covalent character, and hence offer other unique polymorphs to test a^2c. We find rutile-type MgF_2 as the lowest energy amorphous-accessible phase, mostly consistent with annealing of films deposited above -50 °C (where a slightly different $CaCl_2$ phase was observed prior to rutile).[19] Metastable β form of InCl was reported to nucleate on annealing of a-InCl deposited at low temperatures, instead of the stable distorted-rock salt α form,[20] as predicted in this work. Aouni and Bauer-Grosse[21] discovered the orthorhombic carbide Mn_2C by crystallizing a sputtered amorphous film. They identified the structure to be of ζ-Fe_2N type, in line with the a^2c prediction. For GeTe, a^2c yields the NaCl-type high-temperature β phase, which crystallizes out of a-GeTe instead of the ground state *R3m* phase.[22] Despite the presence of a stable Li_3N phase, the analogous Na_3N phase remained elusive for years.[23] Fischer and Jansen made this highly unstable compound in the anti-ReO_3 structure by crystallizing an amorphous precursor deposited on a liquid nitrogen cooled substrate to lock in a highly atomized Na-N mixture.[23] In a^2c, the reported deposition temperature (77 K) was essential for limiting the development of short-range order and dimerization in the amorphous precursor, and in turn for crystallization of anti-ReO_3-type Na_3N. This result is not only validating a^2c but also the ability of tuning the amorphous structure to induce a target.

When polytypes or disorder are at play, a^2c predictions require further discussion. $YbFeO_3$ adopts an orthorhombic perovskite structure in its stable form, but Nishimura et al.[24] synthesized a metastable hexagonal polymorph ($P6_3cm$) from an amorphous precursor, where Fe forms an unusual trigonal bipyramidal coordination with five oxygen atoms, stacked between layers of

closed-packed [YbO_6]. With a^2c, we find a closely-matching polytype in *R3m* that deviates from the original report only in its stacking sequence, hence is nearly isostructural (Fig. 2). Amorphous Al_2O_3 goes through topotactically-related transition aluminas on annealing, such as gamma (γ) and theta (θ) phases, before reaching α-corundum phase. γ and θ phases differ mainly by the degree of site disorder and the ratio of Al^{3+} occupying the tetrahedral and octahedral sites of the cubic-close-packed O^{2-} sublattice. The structure of the γ phase is still debated,[25] whereas θ phase is considered a similar but more ordered monoclinic variant. In a-Al_2O_3 films, while γ phase was historically suggested to crystallize first, recent experiments refined the product to be θ phase,[26] and showed θ phase has the closest structural alignment with amorphous alumina among all polymorphs.[27] The prediction from a^2c is consistent with these recent reports.[26,27]

Supplementary Note 2

In Supplementary Fig. 1, the selected subcell starts with suboptimal Ti-Ti and O-O separations and high energy due to periodicity constraint, but the local Ti-O-Ti motif present at ~150° remains as the backbone towards anatase as the cell steadily relaxes to lower energies by local atomic movements and distortions, exclusively on a downhill path. The suboptimal bonds, which would be local excitations activated thermally in annealing, resolve early: first Ti-Ti and then O-O peaks disappear in the first 40 steps. Beyond this point, structural fingerprints of the cell remain close to that of the amorphous state in both radial and bond angle distribution functions, and Ti centers of the backbone rotate to lock into the anatase arrangement. The peak positions and intensities in distribution functions of the final crystal align with their counterparts in the a-TiO_2. In fact, in all systems we studied, the structural fingerprint of the product aligned well with the amorphous parent (Supplementary Fig. 2).

Supplementary Note 3

In Extended Data Fig. 1, the first crystal to appear is predicted as Fe_4B, requiring no long-range transport. Such a transitory phase was reported as super-saturated α-Fe(B) or Fe_4B, but its structure has not been established beyond having bcc-like or layered-like features.[28–30] In agreement, we find the Fe_4B phase has distorted bcc-Fe layers separated by layers of tricapped trigonal [BFe_9] prisms. Interestingly, this pattern extends to lower Fe contents (the phases highlighted as dark blue squares towards Fe) forming a homologous series. The well-known metastable crystallization product Fe_3B (*Pnma*)[31] is found exactly by a^2c, which can be viewed as an extension of the series where no bcc-Fe layers remain in the structure. The ground state

tetragonal Fe_2B (I4/m) and bcc-Fe phases are also found, which implies their crystallization is bottlenecked by long-range diffusion.

Supplementary Note 4

Due to the multivalent nature of their cations, the synthesis and crystallization of amorphous Ti or V oxide precursors or films are highly complex to handle, both experimentally and computationally. The crystallization products in these systems are highly sensitive to experimental conditions.[10,32–34] For example, amorphous TiO_2 often crystallizes into anatase and in certain cases into rutile,[10,14,35] with recent thin films studies accessing even brookite.[32–34] As discussed by Petkov et al.[10] on p.27 (and references 36-38 therein) and as in Refs. 13 and 14, brookite was not a common crystallization product for a-TiO_2, but recent thin film studies revealed its emergence under certain deposition conditions, which is discussed separately in the main text and Supplementary Note 6.

In this section, we focus on the phase selection problem in amorphous TiO_2 using the methods developed as part of a^2c, and aim to gain insights into experimental observations, by drawing parallels to the influence of preparation conditions of the amorphous precursor in MQMD, and the resulting changes in the amorphous structures on crystallization products in a^2c. Methodology is described in Supplementary Methods.

In Supplementary Figs. 6a and 6b, we show that across practically accessible molecular simulation times or at various quench temperatures, there are only subtle changes in the mean Ti-Ti, Ti-O and O-O pair correlations representing the resulting amorphous structures. This observation is similar to prior molecular simulations of glassy systems.[36,37] We observe slower quenches to introduce slightly more definition in the Ti-Ti ordering (Supplementary Figures 6a and 8). Overall, when these batches of amorphous samples generated with different quench times or quench temperatures are subjected to crystallization in a^2c, they all find anatase and rutile, and on average yield similar ratios of anatase to rutile among the crystallized subcells (Supplementary Figures 6c and 6d). Both of these phases are known to crystallize competitively and sometimes concurrently from amorphous TiO_2, hence a^2c finding these two phases predominantly (>97% as exemplified in Supplementary Fig. 3) is qualitatively in line with the experiments.

The next question to tackle is why under certain experimental conditions the anatase-to-rutile ratio may be skewed from one phase to the other. Interestingly, on an individual level, there are

a-TiO_2 samples which yield notably higher or lower anatase:rutile ratios than others (Supplementary Figure 7). We observe that the first peaks in Ti-Ti RDFs of amorphous samples with higher and lower ratios of anatase-to-rutile among their crystallized subcells, deviate in opposite directions from the mean Ti-Ti RDF of amorphous samples generated at their respective conditions. In these RDFs, we find that Ti-Ti separations that are more commensurate with edge sharing TiO_x (~3 Å) become relatively more intensified for the higher anatase:rutile ratio cases, whereas the separations commensurate with vertex-sharing intensify in lower anatase:rutile ratio cases (~3.5 Å). Since anatase has twice as many edge sharing Ti-centers than rutile, these findings reveal a connection between the nature of the Ti-Ti ordering in amorphous precursors and the emergence of anatase or rutile (or their relative proportions), providing a plausible explanation for the sensitivity of the products to experimental conditions. For example, while access to much longer timescales are impractical in simulations, the trend observed in Supplementary Fig. 8 indicates that at experimental time scales commensurate with slower deposition of amorphous TiO_2 films, the first hump in the Ti-Ti ordering would further strengthen, promoting anatase further, as observed in experiments.[33,34] A similar structural argument was arrived at by Petkov et al.[10] via Reverse Monte Carlo simulations of experimental measurements across a-TiO_2 synthesized using a range of methods. See Supplementary Note 6 for further discussion of the case of brookite.

Supplementary Note 5

All-atom amorphous TiO_2 || polymorph interface simulations are carried out as described in Supplementary Methods. Using the minimum interfacial energy found among all samples of each termination among distributions shown in Supplementary Fig. 9, Wulff-area weighted amorphous-TiO_2 || polymorph interfacial energies are estimated as 0.68, 0.72 and 0.91 J/m^2 for anatase, rutile and brookite respectively. Examples of these low energy interface configurations are shown in Supplementary Fig. 10 for each polymorph.

Supplementary Note 6

As discussed in Supplementary Note 4, brookite is not a common crystallization product for a-TiO_2, but recent thin film studies indicate it can emerge at certain film thicknesses, deposition rates and oxygen partial pressures (or oxygen sub-stoichiometry in the films).[33,34] In particular, Agirseven et al.[34] showed brookite emerges in a narrow O_2 pressure (or Ti:O sub-stoichiometry) and film thickness envelope (their Fig. 2), hence stabilized exclusively by conditions peculiar to such highly thin films. In relatively thin films, surface and interface effects would dominate

stability, and the amorphous materials can even be thermodynamically stabilized with respect to crystalline polymorphs.[38] To investigate if sub-stoichiometry in TiO_x may affect the products (as uncovered in Refs 33 and 34) in a^2c, we ran a^2c at x~1.8 and 2 with more stringent settings (n_{grid} = 20 and n_{max}= 24) than in Extended Data Table 1, and did not encounter brookite among products. We further ran all-atom interface simulations with amorphous TiO_x at x~1.8 and these TiO_2 polymorphs, analogous to the simulations in Supplementary Figure 9, and found that the sub-stoichiometric amorphous form renders most interface terminations reactive, yielding negative interfacial energies due to spontaneous progression of the crystals at the expense of the amorphous film. Hence the crystallization of brookite or other phases from the sub-stoichiometric a-TiO_x does not fit the classical view of nucleation as-is, and requires complex and realistic simulations of the nucleation process in the film, remaining an open challenge for computational modeling.

Supplementary Note 7

We confirm the representativeness of the amorphous structures generated by our MQMD workflow that uses our universal GNoME interatomic potential by comparing the reduced partial radial distribution functions (RDFs) of amorphous atomic configurations of Al_2O_3, $Fe_{80}B_{20}$ and TiO_2 to the same functions extracted from experimental measurements in Supplementary Figure 11.[10–12] The experiment-derived RDFs are obtained via reverse Monte Carlo (RMC) simulations of diffraction data in TiO_2 and Al_2O_3 and deconvolution of isotope substituted neutron and X-ray diffractions for $Fe_{80}B_{20}$ in respective studies. Hence experimental RDFs inherently have uncertainties stemming from the limited Q-resolution or scattering power in diffractions, compositional fluctuations or the nature and assumptions of RMC simulations (which are discussed in cited references). We observe that the partial RDFs, including near neighbor and second peak separations, agree reasonably well with experiment-derived RDFs, on par with prior computational studies. For example, Nold and co-workers[11] performed a series of X-ray and neutron diffraction experiments on $Fe_{80}B_{20}$ glasses with isotopic substitutions that yielded three total structure factors with different Fe/B scattering ratios, which were then used to solve for the Fe-Fe, Fe-B and B-B partial structure factors and finally Fourier transformed to obtain the displayed RDFs. Our results capture the nature of the short and medium range order present in their RDFs for Fe-B and Fe-Fe, and as noted in their paper, the experimental B-B result is prone to high uncertainty. In fact, follow-up work[39–41] argued that some degree of B-B neighboring is expected, in further agreement with the additional peak observed in our RDFs. Agreement is generally good for all three of M-O, M-M and O-O (M=Al,Ti) in Al_2O_3 and TiO_2. There is a

characteristic splitting in the first peak of Ti-Ti, which is captured in our model as well, but both in our simulations) and in the RMC simulations of samples prepared with different conditions by Petkov et al.,[10] there is some variability in relative intensities of these splitting peaks depending on the samples. Hence overall, we conclude that the amorphous samples generated with our MQMD framework and GNoME potential are reasonable approximations to the real-world amorphous materials, but of course, structural details and hence crystallization product can have a dependence on the synthesis process yielding the amorphous precursor.

Supplementary Note 8

Since a^{2}c is focused on identifying the polymorphs of individual systems, the performance of our GNN potential in capturing the relative stabilities of polymorphs would also be a strong indicator of its predictive performance. We calculated the relative stabilities of polymorphs in various systems listed in Figure 2, for which there were at least two polymorph structures within a broad energy range of 0.2 eV/atom above the lowest energy polymorph at the given compositions in the Materials Project database. Density functional theory (DFT) calculations were performed as part of our GNoME framework and are consistent with the standard MP2020 settings available in pymatgen.[8] The reference polymorph in each system is selected as that with the lowest DFT energy, against which the relative stabilities of all other polymorphs in the respective system are calculated. As shown in Supplementary Fig. 12, our GNN potential's predictions of relative stabilities are highly accurate, where the mean absolute error reaches 9 meV/atom for the 135 polymorphs shown in the figure. Furthermore, we find that the resulting stability rankings are also accurate, with a Spearman's rank correlation coefficient for each system at 0.8 or above. This high accuracy in predicting relative energies and rank correlations among polymorphs indicates that a large component of the error in energy predictions by the ML potential is compositional (i.e. separable from structure), hence cancels out to some degree in case of polymorphs.

Supplementary References

1. Brunner, G. O. A definition of coordination and its relevance in the structure types AlB_2 and NiAs. *Acta Crystallogr. A* **33**, 226–227 (1977).
2. Pan, H. *et al.* Benchmarking Coordination Number Prediction Algorithms on Inorganic Crystal Structures. *Inorg. Chem.* **60**, 1590–1603 (2021).
3. Nikaido, Y. *et al.* Diffusion Monte Carlo Study on Relative Stabilities of Boron Nitride Polymorphs. *J. Phys. Chem. C* **126**, 6000–6007 (2022).
4. Jain, A. *et al.* Commentary: The Materials Project: A materials genome approach to accelerating materials innovation. *APL Mater.* **1**, 011002 (2013).
5. Amsler, M., Hegde, V. I., Jacobsen, S. D. & Wolverton, C. Exploring the High-Pressure Materials Genome. *Phys. Rev. X* **8**, 041021 (2018).
6. Sun, W. & Ceder, G. Efficient creation and convergence of surface slabs. *Surf. Sci.* **617**, 53–59 (2013).
7. Tran, R. *et al.* Surface energies of elemental crystals. *Sci Data* **3**, 160080 (2016).
8. Ong, S. P. *et al.* Python Materials Genomics (pymatgen): A robust, open-source python library for materials analysis. *Comput. Mater. Sci.* **68**, 314–319 (2013).
9. Shinozaki, K., Hashimoto, K., Honma, T. & Komatsu, T. TEM analysis for crystal structure of metastable $BiBO_3$ (II) phase formed in glass by laser-induced crystallization. *J. Eur. Ceram. Soc.* **35**, 2541–2546 (2015).
10. Petkov, V., Holzhuter, G., Troge, U., Gerber, T. & Himmel, B. Atomic-scale structure of amorphous TiO_2 by electron, X-ray diffraction and reverse Monte Carlo simulations. *Journal of Non-Crystalline Solids* **231**, 17–30 (1998).
11. Nold, E., Lamparter, P., Olbrich, H., Rainer-Harbach, G. & Steeb, S. Determination of the partial structure factors of the metallic glass $Fe_{80}B_{20}$. *Z. Naturforsch. A: Phys. Sci.* **36**, 1032–1044 (1981).

12. Lamparter, P. & Kniep, R. Structure of amorphous Al_2O_3. *Physica B* **234-236**, 405–406 (1997).

13. Matthews, A. The crystallization of anatase and rutile from amorphous titanium dioxide under hydrothermal conditions. *American Mineralogist* **61**, 419–424 (1976).

14. Abbasi, M. *et al.* In situ observation of medium range ordering and crystallization of amorphous TiO_2 ultrathin films grown by atomic layer deposition. *APL Mater.* **11**, 011102 (2023).

15. Bettermann, P. & Liebau, F. The transformation of amorphous silica to crystalline silica under hydrothermal conditions. *Contrib. Mineral. Petrol.* **53**, 25–36 (1975).

16. Stone, K. H. *et al.* Influence of amorphous structure on polymorphism in vanadia. *APL Mater.* **4**, 076103 (2016).

17. Ebralidze, I., Lyahovitskaya, V., Zon, I., Wachtel, E. & Lubomirsky, I. Anomalous pre-nucleation volume expansion of amorphous $BaTiO_3$. *J. Mater. Chem.* **15**, 4258–4261 (2005).

18. Lyahovitskaya, V. *et al.* Formation and thermal stability of quasi-amorphous thin films. *Phys. Rev. B* **71**, 094205 (2005).

19. Bach, A. *et al.* Structural Evolution of Magnesium Difluoride: from an Amorphous Deposit to a New Polymorph. *Inorg. Chem.* **50**, 1563–1569 (2011).

20. Bach, A., Fischer, D. & Jansen, M. Metastable Phase Formation of Indium Monochloride from an Amorphous Feedstock. *Z. Anorg. Allg. Chem.* **639**, 465–467 (2013).

21. Bauer-Grosse, E. & Aouni, A. New ζ-Mn_2C hemicarbide formed during the crystallization of an amorphous sputtered $Mn_{1-x}C_x$ film. *J. Alloys Compd.* **336**, 190–195 (2002).

22. Nonaka, T., Ohbayashi, G., Toriumi, Y., Mori, Y. & Hashimoto, H. Crystal structure of GeTe and $Ge_2Sb_2Te_5$ meta-stable phase. *Thin Solid Films* **370**, 258–261 (2000).

23. Fischer, D. & Jansen, M. Synthesis and Structure of Na_3N. *Angew. Chem. Int. Ed.* **41**, 1755–1756 (2002).

24. Nishimura, T., Hosokawa, S., Masuda, Y., Wada, K. & Inoue, M. Synthesis of metastable rare-earth–iron mixed oxide with the hexagonal crystal structure. *J. Solid State Chem.* **197**, 402–407 (2013).

25. Ayoola, H. O. *et al.* Evaluating the accuracy of common γ-Al_2O_3 structure models by selected area electron diffraction from high-quality crystalline γ-Al_2O_3. *Acta Mater.* **182**, 257–266 (2020).

26. Broas, M., Kanninen, O., Vuorinen, V., Tilli, M. & Paulasto-Kröckel, M. Chemically Stable Atomic-Layer-Deposited Al_2O_3 Films for Processability. *ACS Omega* **2**, 3390–3398 (2017).

27. Pugliese, A. *et al.* Atomic-Layer-Deposited Aluminum Oxide Thin Films Probed with X-ray Scattering and Compared to Molecular Dynamics and Density Functional Theory Models. *ACS Omega* **7**, 41033–41043 (2022).

28. Duhaj, P. & Svec, P. Formation of metastable phases from amorphous state. *Materials Science and Engineering A* **226228**, 245–254 (1997).

29. Köster, U. & Herold, U. Crystallization of amorphous $Fe_{80}B_{20}$. *Scr. Metall.* **12**, 75–77 (1978).

30. Aykol, M., Mekhrabov, A. O. & Akdeniz, M. V. Nano-scale phase separation in amorphous Fe–B alloys: Atomic and cluster ordering. *Acta Mater.* **57**, 171–181 (2009).

31. Duhaj, P., Švec, P. & Zemčík, T. Micromechanism of crystallization of $Fe_{80}B_{20}$ amorphous alloy. *Mater. Lett.* **9**, 235–241 (1990).

32. Haggerty, J. E. S. *et al.* High-fraction brookite films from amorphous precursors. *Sci. Rep.* **7**, 15232 (2017).

33. Mangum, J. S. *et al.* Selective brookite polymorph formation related to the amorphous precursor state in TiO_2 thin films. *J. Non-Cryst. Solids* **505**, 109–114 (2019).

34. Agirseven, O. *et al.* Crystallization of TiO_2 polymorphs from RF-sputtered, amorphous thin-film precursors. *AIP Adv.* **10**, 025109 (2020).

35. Lin, C.-P., Chen, H., Nakaruk, A., Koshy, P. & Sorrell, C. C. Effect of Annealing Temperature on the Photocatalytic Activity of TiO_2 Thin Films. *Energy Procedia* **34**, 627–636 (2013).

36. Pandy, Scherich & Drabold. Density functional theory model of amorphous zinc oxide (a-ZnO) and a-$X_{0.375}Z_{0.625}O$ (X= Al, Ga and In). *J. Non-Cryst. Solids* **455**, 98–101 (2017).

37. Pan, S. P., Qin, J. Y., Wang, W. M. & Gu, T. K. Origin of splitting of the second peak in the pair-distribution function for metallic glasses. *Phys. Rev. B* **84**, 092201 (2011).

38. Aykol, M. & Persson, K. A. Oxidation Protection with Amorphous Surface Oxides: Thermodynamic Insights from Ab Initio Simulations on Aluminum. *ACS Appl. Mater. Interfaces* **10**, 3039–3045 (2018).

39. Arime, H. *et al.* Structural Study of $Fe_{80}B_{20}$ Amorphous Alloy by Anomalous X-ray Scattering Coupled with Neutron Diffraction. *JPS Conf. Proc.* **8**, 031019 (2015).

40. Yang, L., Qin, J., Pan, S. & Bian, X. The shoulder in the second peak of the pair correlation function of superheated liquid $Fe_{80}B_{20}$ alloy. *J. Non-Cryst. Solids* **357**, 3207–3211 (2011).

41. Ganesh, P. & Widom, M. *Ab initio* simulations of geometrical frustration in supercooled liquid Fe and Fe-based metallic glass. *Phys. Rev. B* **77**, 014205 (2008).
